# Data Processing of Product Ion Spectra: Quality Improvement by Averaging Multiple Similar Spectra of Small Molecules

**DOI:** 10.5702/massspectrometry.A0106

**Published:** 2022-12-15

**Authors:** Fumio Matsuda, Shuka Komori, Yuki Yamada, Daiki Hara, Nobuyuki Okahashi

**Affiliations:** 1 Graduate School of Information Science and Technology, Osaka University, Osaka, Japan; 2 Osaka University Shimadzu Omics Innovation Research Laboratories, Osaka University, Osaka, Japan

**Keywords:** metabolomics, precision, elemental composition search, data-dependent acquisition mode

## Abstract

In metabolomics studies using high-resolution mass spectrometry (MS), a set of product ion spectra is comprehensively acquired from observed ions using the data-dependent acquisition (DDA) mode of various tandem MS. However, especially for low-intensity signals, it is sometimes difficult to distinguish artifact signals from true fragment ions derived from a precursor ion. Inadequate precision in the measured *m*/*z* value is also one of the bottlenecks to narrowing down the candidate compositional formula. In this study, we report that averaging multiple product ion spectra can improve *m*/*z* precision as well as the reliability of fragment ions that are observed in such spectra. A graph-based method was applied to cluster a set of similar spectra from multiple DDA data files resulting in creating an averaged product-ion spectrum. The error levels for the *m*/*z* values declined following the central limit theorem, which allowed us to reduce the number of candidate compositional formulas. The improved reliability and precision of the averaged spectra will contribute to a more efficient annotation of product ion spectral data.

## INTRODUCTION

In metabolomics studies using high-resolution mass spectrometry (MS), a precise mass-to-charge ratio (*m*/*z*) value is measured to deduce the compositional formulae of a metabolite-derived ion.^1)^ In addition, the product ion spectrum of the detected ion is simultaneously acquired using the data-dependent acquisition (DDA) mode of various tandem MS, such as quadrupole-time-of-flight (Q-TOF) MS.^2–7)^ The fragmentation pattern in the product ion spectrum is also used to annotate structural information regarding the metabolite. In the DDA mode, a full-scan mass spectrum is obtained without fragmentation (MS1 scan), from which the set of the most abundant ions is selected as the precursor ion to obtain the product ion spectra. The DDA mode algorithm selects precursor ions that increase metabolite coverage by avoiding the acquisition of redundant data from an identical precursor ion.^8)^ Thus, data obtained in the DDA mode often contain artifact signals that are generated during automated data acquisition. Natural and electronic noise can also produce artifact signals in product ion spectra. Data interpretation is sometimes difficult because true fragment ions derived from a precursor ion are barely distinguishable from artifact signals, particularly for the case of low-intensity signals. Moreover, even for a true fragment ion, inadequate precision in the measured *m*/*z* value is one of the bottlenecks to deducing candidate compositional formula.^9–11)^

In the classical product ion scan mode, a set of product ion spectra is iteratively obtained from an identical target precursor ion in one data acquisition run. These closely similar spectra are then integrated into an averaged product ion spectrum so as to improve the signal-to-noise ratio (*S*/*N*) level. The averaged product ion spectrum typically has better precision in terms of *m*/*z* value than that of the original spectra before averaging. In addition, metabolite-derived signals can be distinguished more reliably from other noise signals.

Similar averaging is also possible for metabolomics DDA data, as has been performed for shotgun proteomics data and is available in metabolomics tools such as MZmine3, OpenMS, CSI:FingerID, and MS-FINDER FSEA.^12–16)^ In most metabolomic studies, a set of data files is acquired from multiple biological samples with similar metabolite profiles.^17–22)^ Many of the available software tools have peak-picking functions that allow metabolite signals in the chromatogram of MS1 scan data to be identified. Recently developed peak-picking software tools can be used to identify a common metabolite signal among multiple DDA data files and calculate the mean *m*/*z* value of the precursor ions.^23)^ Furthermore, each DDA data file will include similar product ion spectra when multiple DDA data files are obtained from similar biological samples. In this study, two product ion spectra were considered to be similar based on the similarity of their spectra patterns and the *m*/*z* values of the precursor ions. Using similarity information, we were able to directly cluster a population of closely similar product ion spectra without the need for a peak-picking process. When many similar product ion spectra are observed reproducibly among many data files, this suggests that the observed product ion spectra are reliable with a lower probability of coincidence. Moreover, a higher *S*/*N* ratio can be obtained by averaging a larger number of very similar data. Thus, it would be expected that the levels of *m*/*z* precision and the reliability of product ion observations would likely be improved by gathering a larger number of spectra. However, the degree of improvement obtained by averaging has not yet been thoroughly investigated.

In this study, we demonstrated that the levels of *m*/*z* precision and the reliability of fragment ions can be improved by averaging the product ion spectra. For this purpose, we used 94 DDA data files from yeast lipidomic studies. The lipidomics dataset is suitable for investigating the degree of improvement because the exact answers are available owing to the intensive annotation of most product ions of lipids.^24)^ It should be noted that the purpose of this study was not the lipidomic profiling of yeast or the annotation of the novel metabolites. To average the product ion spectra, a graph-based method was used to create clusters of closely similar product ion spectra from multiple DDA data files. The results of this study demonstrate that the improvements follow the central limit theorem.

## EXPERIMENTAL PROCEDURES

### Preparation of the lipidomics dataset

This study used 94 lipidomics data files obtained from three distinct *Saccharomyces cerevisiae* research projects over a period of two years (Supplementary Table 1). Details of the *S. cerevisiae* strains and their cultivation conditions have been reported elsewhere. All data files were obtained from *S. cerevisiae* cells that were cultured under similar conditions using an identical data acquisition method.^25)^ Various *S. cerevisiae* strains were cultured in a synthetic dextrose (SD) medium (5 g/L glucose, 6.7 g/L yeast nitrogen base without amino acids (Difco Laboratories, Detroit, MI, USA)). The main cultures were performed using 50 mL of SD medium (5 g/L) in a 200 mL baffled flask with an initial OD_600_ of 0.05 as a preculture and incubated at 10–30°C with an agitation speed of 120 rpm. In most cases, the cell broth (OD_600_=1.0) was collected and used for an identical lipidomic analysis, as described in a previous study.^25)^ Briefly, the lipid fraction was extracted *via* the chloroform–methanol–water method and then used for a liquid chromatography (LC)-quadrupole(Q)-time-of-flight (TOF)-mass spectrometry (MS) analysis in the positive ion mode using the DDA method (LCMS-9030, Shimadzu, Kyoto, Japan). The parameters were as follows: MS1 and MS2 mass ranges: *m*/*z* 70–1750, MS1 accumulation time: 250 ms, MS2 accumulation time: 66 ms, cycle time: 1240 ms, collision energy: 35 eV, and collision energy spread: 20 eV. The obtained data files were converted into the mzXML format containing the centroid data using the LabSolutions Insight function (Shimadzu).^26)^

### Construction of averaged product ion spectra

All of the data processing procedures were performed using in-house Python3 scripts. An mzXML file was parsed using the xml.etree.ElementTree package. A pair of two product ion spectra data was considered to be similar when the difference in the *m*/*z* and retention time of the precursor ion was between 0.01 and 1.0 min and when the cosine product score of the product ion spectra was greater than 0.9. The cosine (dot) product is a method that is used to evaluate the similarity between two mass spectra, whose score ranges from 0 (no similarity) to 1.0 (identical).^27)^ Two fragment ions were considered to be identical when Δ*m*/*z* was less than 0.01 in the determination of the cosine product score. Here, an averaged product ion spectrum was constructed for a given target lipid, such as the protonated molecule ([M+H]^+^) of PE(34 : 1), whose elemental composition was C_39_H_77_NO_8_P. This study examined lipids in yeast containing C, H, N, O, and P atoms. Moreover, no elemental composition search was conducted to determine the elemental composition of the precursor ion during the construction of the averaged product ion spectrum. Generally, an averaged product ion spectrum is constructed for a given target precursor ion (C_e_H_f_N_g_O_h_P_i_, *m*/*z*=*m*/*z*_theoretical_) using the following procedure:

1) Product ion spectra derived from precursor ions within *m*/*z*_theoretical_ ± 0.02 were collected from 94 DDA data files.

2) A similarity graph was created from the collected data based on similarity level.

3) Clusters of similar product ion spectra were extracted by finding complete graphs (cliques) in the created similarity graph using the NetworkX package (https://networkx.org/).^28)^

4) To obtain an average mass spectrum, all fragment ions were collected from a complete graph (cluster). The total number of product ion spectra in this cluster is denoted as *j*.

5) A similarity graph of all fragment ions was created by considering that two fragment ions with Δ*m*/*z*<0.01 were similar.

6) If the total number of fragment ions (*k*) in a clique is *k*>0.7×*j*, this clique is employed to create an averaged mass spectrum.

7) The median *m*/*z* and relative intensity values of the fragment ions were used for the averaged mass spectra.

8) The *m*/*z* value of the precursor ion of the averaged mass spectrum was determined as the median *m*/*z* value of all precursor ions.

9) The precursor *m*/*z* value of the averaged spectrum (*m*/*z*_measured_) was compared with the theoretical value (*m*/*z*_theoretical_) using the following equation: 
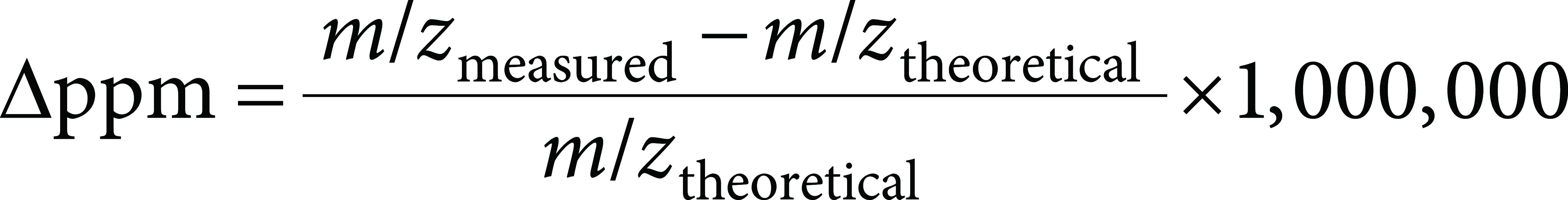
(1) When the Δppm level was less than the threshold level (2 ppm for the precursor ion search), the averaged spectrum was the candidate product ion spectrum derived from the target precursor ion.

10) The elemental composition of each fragment ion in the averaged spectrum was estimated using an elemental composition search. For C_e_H_f_N_g_O_h_P_i_, the upper boundaries of the atom numbers were set to e, f, g, h, and i for C, H, N, O, and P, respectively, with a threshold level of 4.0 ppm. The elemental composition with the lowest Δppm was assigned to the fragment ions.

11) When elemental compositions were successfully assigned to all fragment ions, the averaged spectrum was consistent with that of the target metabolites. The averaged spectrum of the target metabolites was manual curated using the known lipid fragmentation patterns.^24)^

### Elemental composition search

Composition formula searches were performed for a given *m*/*z* value (*m*/*z*_measured_), using the following equation: 
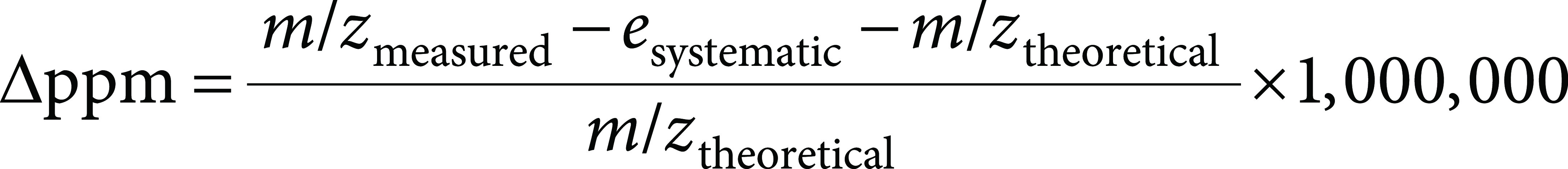
(2)

The threshold of Δppm and value of *e*_systematic_ were set at arbitrary levels. The seven golden rules were employed during searching, except for rule 3 (utilization of isotopic pattern) and rule 7 (removal of chemical derivatization effect).^9)^ The search ranges of the N, P, and S atom numbers were restricted to 0≤N≤3, 0≤P≤2, and 0≤S≤1, respectively, owing to the lipidomics dataset.

## RESULTS

### Bottlenecks in metabolite annotation using product ion spectra obtained by the DDA method

**Figure 1:**
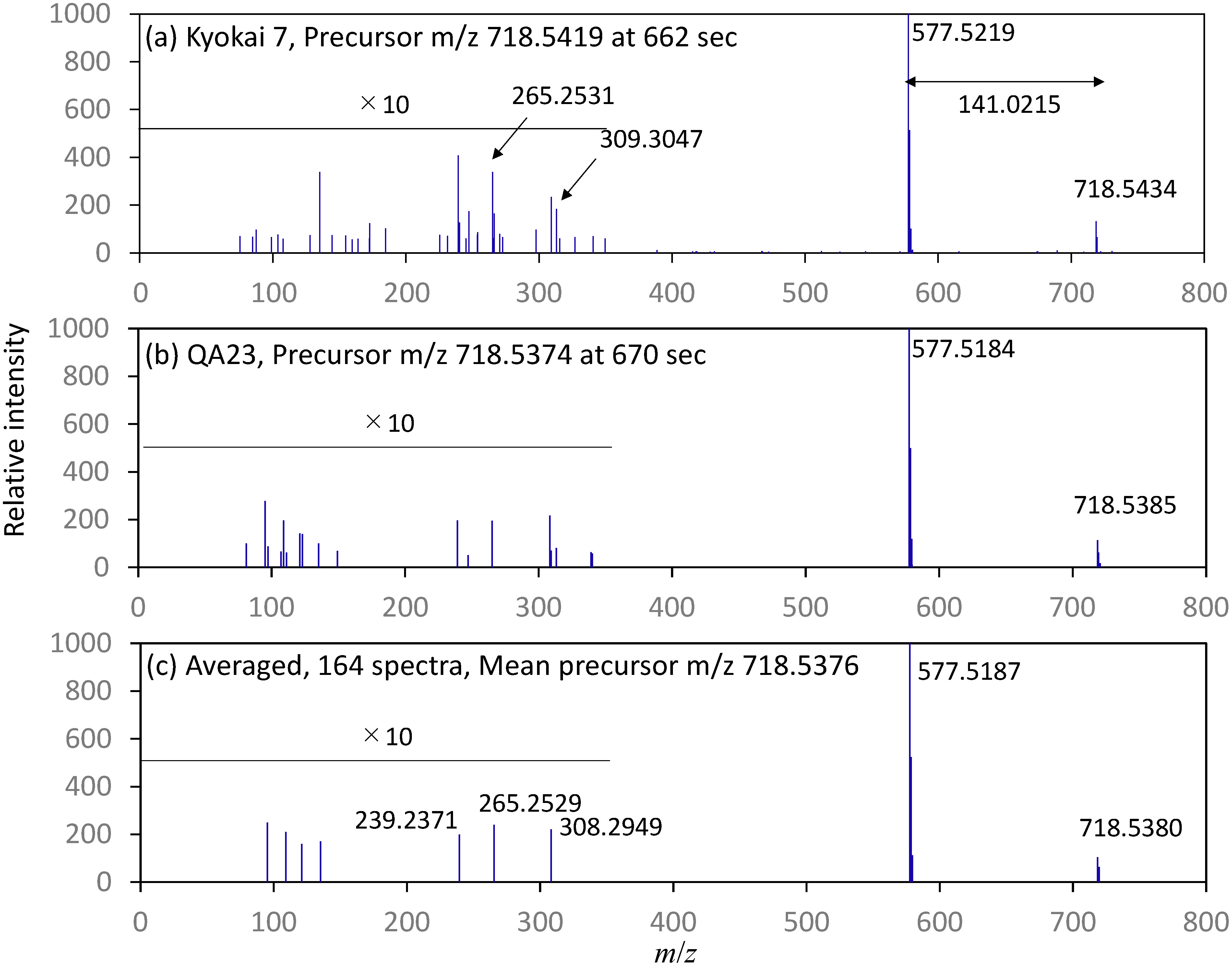
Fig. 1. Comparison between original and averaged product ion spectra of phosphatidylethanolamine (PE) (34 : 1). (a) Original product ion spectrum of a precursor ion with *m*/*z* of 718.5419 at 662 s in DDA data file acquired from Kyokai 7 strains of *S. cerevisiae*. (b) Original ion spectrum of precursor ion with *m*/*z* of 718.5374 at 670 s in another DDA data file for QE23 strain. (c) Averaged product ion spectrum constructed by averaging 164 similar product ion spectra.

Previous lipidomic studies have reported that *S. cerevisiae* contains various phospholipid species.^21,22,29,30)^ For example, a DDA analysis of the Kyokai 7 strain of *S. cerevisiae* in the positive ion mode provided a product ion spectrum from a precursor ion with an *m*/*z* and retention time of 718.5419 and 662 s, respectively (Fig. 1a, Spectrum id: B4_k7_1_pos_7678 in Supplementary Data 1). Notably, the product ion spectrum includes isotope signals owing to the wider window for precursor ion selection employed during the DDA analysis.

Among the fragment ions in the spectrum, the most intense signal at *m*/*z* 577.5219 was likely derived from the analyte. This suggests that the metabolite was phosphatidylethanolamine (PE) (34 : 1), with the compositional formula C_39_H_76_NO_8_P. This was because the measured *m*/*z* value of the precursor ion was similar to the theoretical *m*/*z* value for protonated molecules of PE(34 : 1) ([M+H]^+^, theoretical *m*/*z* 718.5381). Moreover, the neutral loss between the precursor and the most intense fragment ion (141.0215) is in good agreement with the removal of the ethanolamine phosphate ester moiety (C_2_H_8_NO_4_P, theoretical neutral loss of 141.0191), which is a characteristic of PE.^24)^

However, problems were encountered regarding the further analysis of the product-ion spectrum. First, it was unclear whether other weak signals were analyte-related or noise-derived signals, such as *m*/*z* of 265.2531 and 309.3047 with relative intensity levels to the base peak of 3.3% and 2.3%, respectively. Second, there are other possible compositional formulas with narrower Δ*m*/*z*. The error levels between the measured and theoretical *m*/*z* (Δ*m*/*z*) values of the precursor and the most intense fragment ions were 0.0037 (5.2 ppm) and 0.0023 (4.0 ppm), respectively, which were inadequate for narrowing down the candidate formula into a single one.

### Construction of averaged product ion spectra by extraction of similar product ion spectra from multiple DDA data files

**Figure 2:**
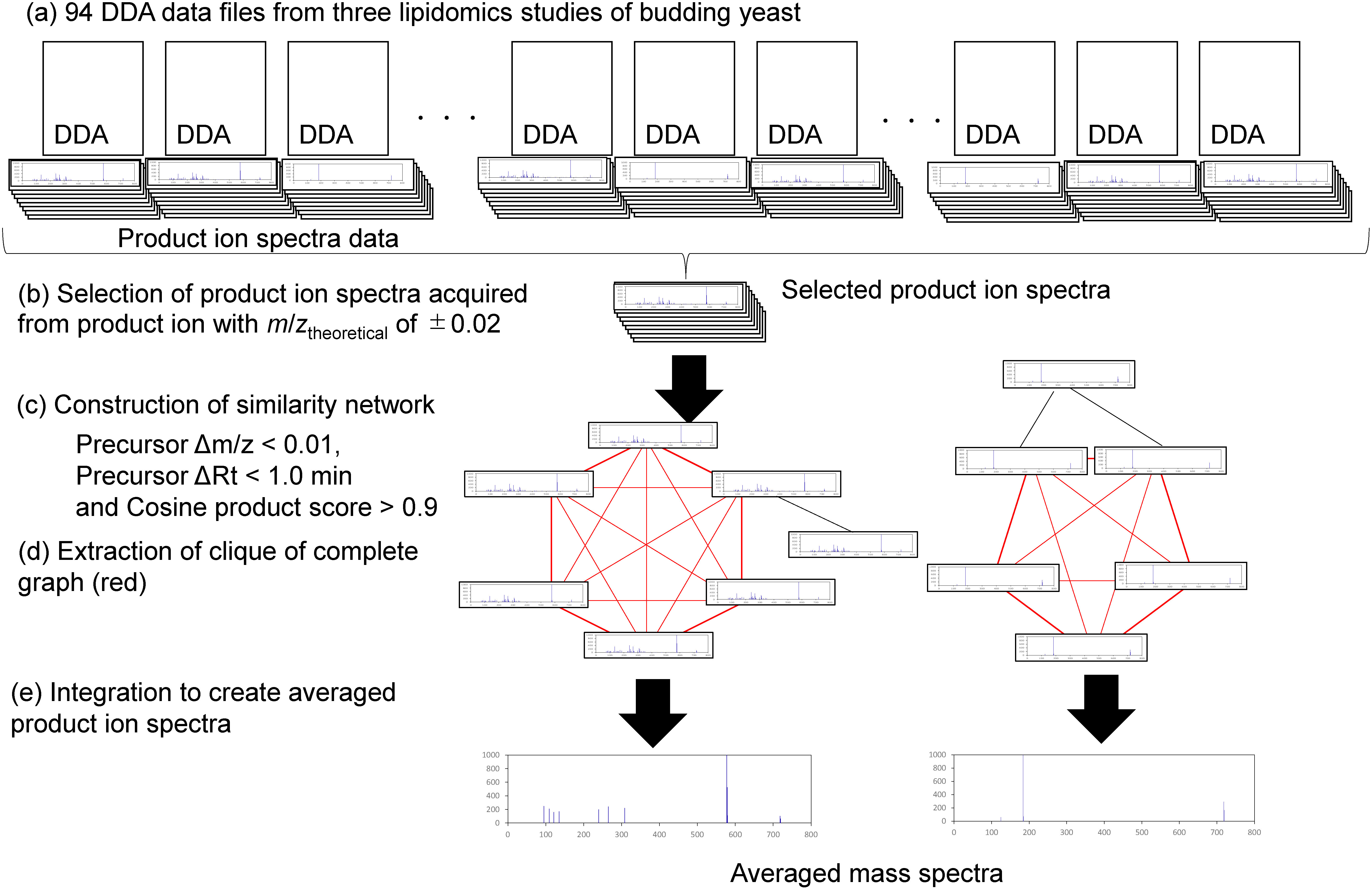
Fig. 2. Construction of averaged product ion spectra from DDA data file. (a) A total of 94 DDA data files were prepared from three lipidomics studies of budding yeast. (b) Product ion spectra derived from precursor ions with *m*/*z*_theoretical_ of ±0.02 were collected from DDA data files. (c) Similarity graph was created among selected product ion spectra data. Pair of two product ion spectra data was considered similar when the difference in *m*/*z* and the retention time of the precursor ion was within 0.01 and 1.0 min. In addition, the cosine product score of product ion spectra was greater than 0.9. (d) Complete graphs (cliques, red lines) were extracted from created similarity graph. The complete graph has edges between all nodes. (e) Averaged spectra of each clique were created by determination of median *m*/*z* of all precursor ions and fragment ions commonly observed in more than 70% of product ion spectra.

To address these problems, we attempted to average multiple similar product ion spectra obtained using the DDA method. In this study, we prepared 94 DDA data files obtained from three distinct yeast lipidomic studies (Fig. 2a, Supplementary Table 1). All data files were acquired by analyzing *S. cerevisiae* samples cultured under similar conditions and using an identical sample preparation and data acquisition protocol using LC-Q-TOF/MS.^25)^ The strains and culture conditions for each study will be reported elsewhere in a future study. In this study, a pair of two product ion spectra data was considered to be similar when the difference in *m*/*z* and retention time of the precursor ion was within 0.01 and 1.0 min and the cosine product score of the product ion spectra was greater than 0.9. For example, another DDA file acquired from the QA23 strain of *S. cerevisiae* included a product ion spectrum similar to that shown in Fig. 1a (Fig. 1b, Spectrum id: QA1_pos_7303 in Supplementary Data 1).

Here, an averaged product ion spectrum was created for protonated molecules of PE (34 : 1) ([M+H]^+^, *m*/*z* of 718.5381) as an example. First, the product ion spectra derived from precursor ions with *m*/*z*=718.5381±0.02 were collected from the 94 DDA data files, which provided a population including 985 product ion spectra (Fig. 2b, Supplementary Data 1). Second, to cluster the population into subpopulations containing closely similar data, a similarity graph was created based on the above-mentioned similarity among the 985 product ion spectra data (Fig. 2c). Third, complete graphs (cliques) were extracted from the similarity graphs (Fig. 2d). A complete graph is one in which every pair of distinct nodes is connected by a unique edge. The clique is more useful than the raw graph because the product ion spectral data in a complete graph are closely similar to each other. Nine cliques containing more than five spectral data were successfully obtained from the similarity graph (Supplementary Data 1).

The largest clique contained 164 spectra, including the data shown in Fig. 1a and b. The total number of product ion signals in the 164 spectra was 8,872, indicating that there were 54.1 product ion signals per product ion spectrum. An averaged spectrum of the 164 spectra in this clique was created, as shown in Fig. 2e. The median *m*/*z* of the 164 precursor ions was determined to be 718.5376, in which the Δ*m*/*z* from the theoretical value was −0.00053 (−0.74 ppm). We employed the median instead of the average throughout this study because of its robustness against outliers. The fragment ions commonly observed in more than 70% of the 164 product ion spectra were then selected, and the median *m*/*z* was determined (see Methods for a detailed procedure).

As a result, an averaged product ion spectrum consisting of twelve product ions was obtained (Table 1a and Fig. 1c). In this study, *n* represents the number of product ion spectral data points used to construct the averaged spectra. These twelve fragment ions appeared to be reliable as analyte-derived ions because they were reproducibly observed among the 164 spectra. The results suggest that the fragment ion of *m*/*z* 265.2529 observed in Fig. 1a can be used for further metabolite annotation. The results also suggest that the raw product ion spectra contain poorly reproducible signals because the number of product ions in the averaged spectra (12) was smaller than that of the raw product ion spectra (54.1 on average). For example, the weak signals in Fig. 1a, such as *m*/*z* 309.3047, should be ignored because of their poor reproducibility. Furthermore, the Δ*m*/*z* for each fragment ion appeared to be significantly lower than that of the original spectrum (Table 1a).

**Table 1:** Table 1. Comparison between measured and theoretical *m*/*z* values in averaged product ion spectra of phosphatidylethanolamine (PE) 34 : 1 and phosphatidylcholine (PC) 31 : 1.

	*m*/*z*_measured_	Relative intensity	Formula	*m*/*z*_theoretical_	Δ*m*/*z*	Δppm
(a) 1st largest clique, *n*=164^1)^ PE(34 : 1) ([M+H]^+^), Fig. 1(c)						
Prec	718.5376		C39H77NO8P	718.5381	−0.00053	−0.74
Frag	95.0852	25	C7H11	95.0855	−0.00030	−3.11
	109.1010	21	C8H13	109.1012	−0.00020	−1.85
	121.1010	16	C9H13	121.1012	−0.00016	−1.30
	135.1166	17	C10H15	135.1168	−0.00019	−1.42
	239.2371	20	C16H31O	239.2369	0.00018	0.75
	265.2529	24	C18H33O	265.2526	0.00029	1.09
	308.2949	22	C20H38NO	308.2948	0.00012	0.40
	577.5187	1000	C37H69O4	577.5190	−0.00039	−0.67
	578.5222	524	C36H69O4 [^13^C]	578.5224	−0.00019	−0.33
	579.5257	112	C37H72O2P	579.5264	−0.00070	−1.21
	718.5380	104	C39H77NO8P	718.5381	−0.00012	−0.17
	719.5413	63	C38H77NO8P[^13^C]	719.5415	−0.00013	−0.18
(b) 2nd largest clique, *n*=64^1)^ PC(31 : 1) ([M+H]^+^)						
Prec	718.5375		C39H77NO8P	718.5381	−0.00059	−0.83
Frag	124.9995	60	C2H6O4P	124.9998	−0.00036	−2.90
	184.0731	1000	C5H15NO4P	184.0733	−0.00022	−1.19
	185.0765	72	C4H15NO4P[^13^C]	185.0767	−0.00011	−0.59
	718.5379	294	C39H77NO8P	718.5381	−0.00028	−0.39
	719.5416	166	C38H77NO8P[^13^C]	719.5415	0.00012	0.17
Average					−0.00020	−0.76
Standard deviation					0.00026	1.09

^1)^ The number of product ion spectral data points used to construct the averaged spectra.

Based on reliability and precision, we can infer that the two fragment ions with *m*/*z* 239.2371 and *m*/*z* 265.2529 were two acyl moieties of 16 : 0 ([C_16_H_31_O]^+^) and 18 : 1 ([C_18_H_33_O]^+^) and that this molecule was deduced to be PE (16 : 0/18 : 1), among other possible structural isomers of PE (34 : 1) (Table 1a). Other product ions, including as *m*/*z* 95.0852 ([C_7_H_11_]^+^), *m*/*z* 109.1010 ([C_8_H_13_]^+^), and *m*/*z* 121.1010 ([C_9_H_13_]^+^), are aliphatic fragments that are commonly generated from various fatty acids.^31)^ The fragment ion at *m*/*z* 308.2949 ([C_20_H_38_NO]^+^) seems to consist of acyl 18 : 1 ([C_18_H_33_O]^+^) and aminoethylene moieties ([CH_2_=CH-NH_2_])). The occurrence of corresponding fragment ions was reported in the product ion spectra of lyso-PEs.^32)^

PC (31 : 1) is a structural isomer of PE (34 : 1) with the identical molecular formula, C_39_H_76_NO_8_P, and known to exist in yeasts.^33)^ We found that the averaged product ion spectra of the second largest clique produced from 64 spectra was PC (31 : 1) (Table 1b). This is because the most intense fragment ion *m*/*z* at 184.0731 was consistent with the choline phosphate ester fragment ion, C_5_H_15_NO_4_P, that is characteristically observed in PC.^24)^ Another characteristic product ion such as a lyso-PC-like structure was not observed owing to the data acquisition condition.^24)^

The averaged product ion spectra of other 7 cliques were also annotated as another structural isomer (phosphatidyldimethylethanolamine (PDME) (32 : 1)) and mixture of these structural isomers (Supplementary Table 2).

These results demonstrate that the averaged spectra can be generated from a population of similar product ion spectra extracted from multiple DDA. Moreover, it was also shown that the improved reliability and precision of the integrated spectra could be the basis for a more detailed metabolite annotation.

### Improvement of precision in *m*/*z* measurement by averaging

Table 1 also reveals that the standard deviation level of the Δ*m*/*z* among the 23 data points was 0.00026, which represents the precision level of the averaged *m*/*z* value. To investigate the relationship between Δ*m*/*z* and *n*, the standard deviation of the Δ*m*/*z* of the averaged spectra was determined on a large scale and compared with that of the original data. For this purpose, we prepared a list of 100 known lipids that have been observed in a *S. cerevisiae* lipidomics studies (Supplementary Table 3, in preparation). The procedure described in the previous section was performed for 100 compositional formulas of [M+H]^+^ or [M+NH_4_]^+^ for each lipid. Finally, 100 averaged product ion spectra of 100 known lipids were constructed from the 6,687 original product ion spectra, in total. The number of raw product ion spectra in the cliques of target lipids, total number of product ions in these spectra, and their product ion/spectra ratios are shown in Supplementary Table 4. All averaged product ion spectra of 100 known lipids and those MassBank record files were presented in Supplementary Data 2 and Spectrum Data 1, respectively.

**Figure 3:**
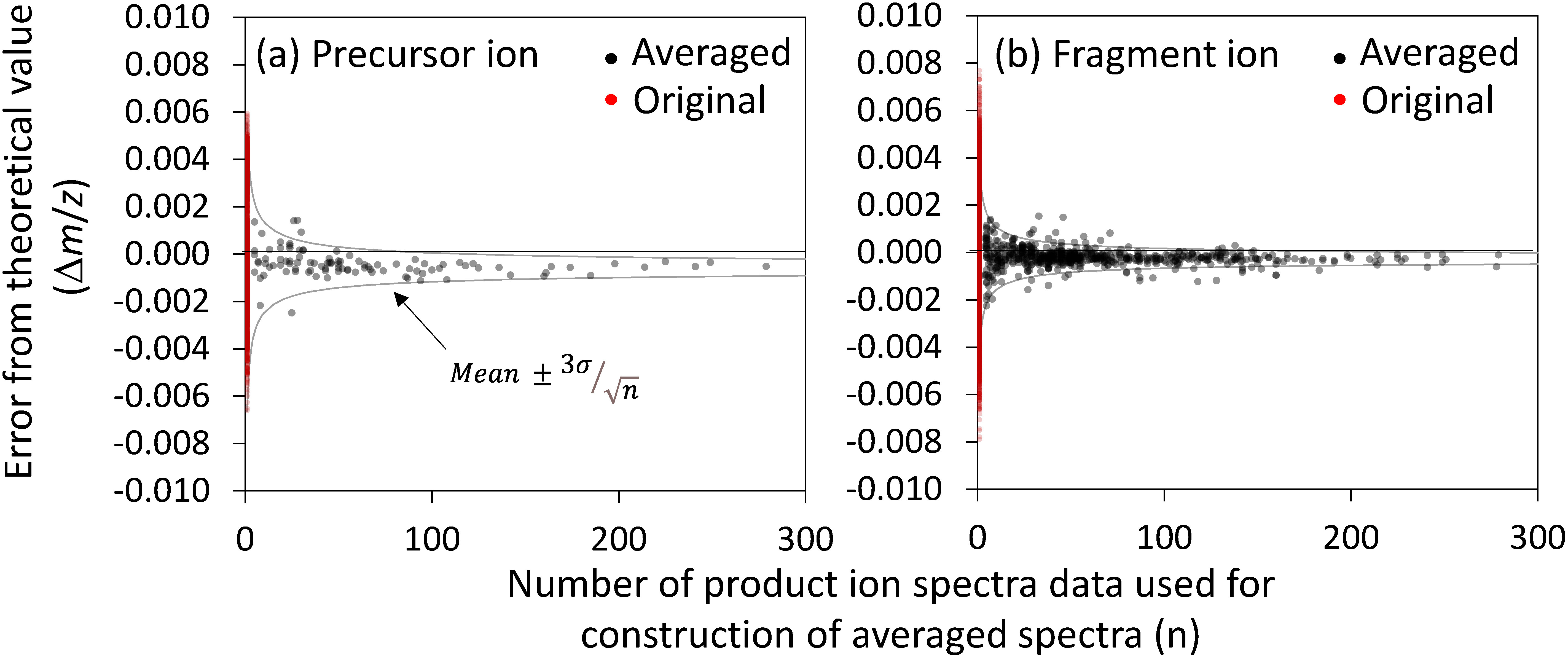
Fig. 3. Relationship between error level in *m*/*z* measurement (Δ*m*/*z*) and number of product ion spectra used for construction of averaged spectra (*n*). Error between measured and theoretical *m*/*z* values in original and averaged product ion spectra of 100 lipid species. Results for 100 precursor ions (a) and 616 fragment ions (b) in 100 averaged data are represented in black. Δ*m*/*z* for 6,687 precursor ions and 39,011 fragment ions in 6,687 original data are shown in red at *n*=1. Black lines indicate ranges of 
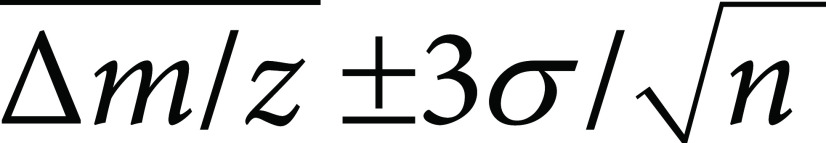
.

First, the Δ*m*/*z* was examined for the precursor ions from the original data of the 6,687 product ion spectra. The standard deviation of the Δ*m*/*z* was determined to be 0.00203 (Table 2 and Fig. 3). In contrast, the standard deviation level of the 100 averaged data constructed above was 0.00057, which is 28% of the original data. The standard deviation was further reduced by averaging a larger number of product ion spectra. As shown in Table 2 and Fig. 3a, the standard deviation level was 0.00023 for the 48 averaged data of *n*>50, which is 11% of that of the original data. A similar reduction in the standard deviation was also observed when the Δppm was used instead of the Δ*m*/*z* (Table 2 and Fig. 3). We also found systematic errors, in addition to random errors, in the measured *m*/*z*. This was because the median Δ*m*/*z* values were approximately −0.0005 both before and after averaging (Table 2 and Fig. 3).

**Table 2:** Table 2. Summary of error between measured and theoretical *m*/*z* values in original and averaged product ion spectra of 100 known lipid species of yeasts. Error levels were shown *via* Δ*m*/*z* and Δppm.

	Precursor ion			Fragment ion		
	Original	Averaged	Averaged (*n*>50)	Original	Averaged	Averaged (*n*>50)
Number of data points	6,687	100	48	39011	616	299
Std (Δ*m*/*z*)	0.00203	0.00057	0.00023	0.00139	0.00042	0.00025
Median (Δ*m*/*z*)	−0.00056	−0.00047	−0.00059	−0.00025	−0.00022	−0.00025
Std (Δppm)	2.71	0.76	0.29	5.18	1.52	1.30
Median (Δppm)	−0.79	−0.66	−0.78	−0.99	−0.73	−0.86

The central limit theorem establishes that the mean value follows a normal distribution, followed by 
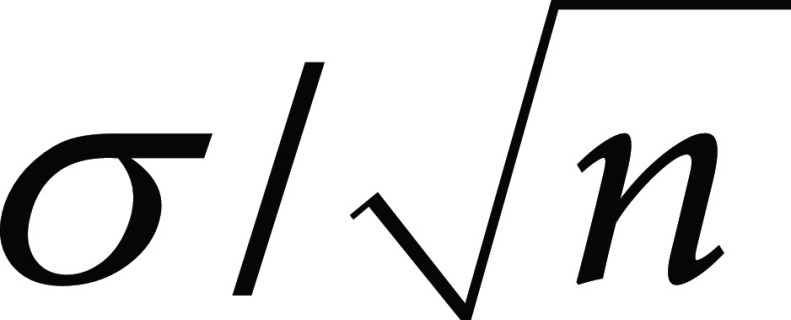
 when the number of random variables *n* following the normal distribution with standard deviation σ is summed up. The central limit theorem also indicates that the distribution of the Δ*m*/*z* of averaged mass spectra should follow a normal distribution and that 99.7% of the Δ*m*/*z* values are inside of the 
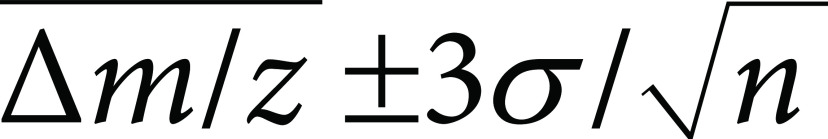
 range.^34)^ Here, 
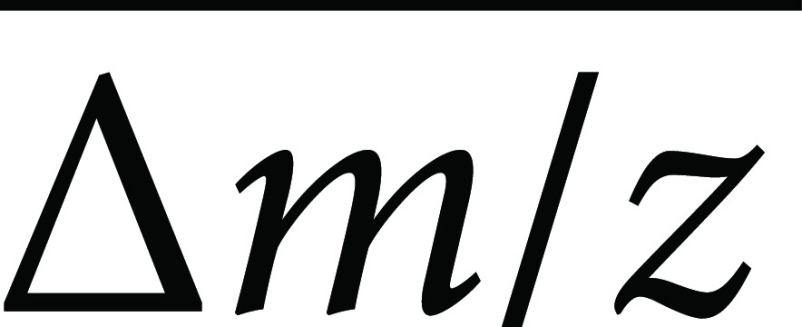
 and σ indicate the mean and standard deviation of the Δ*m*/*z* of the original data. Using 
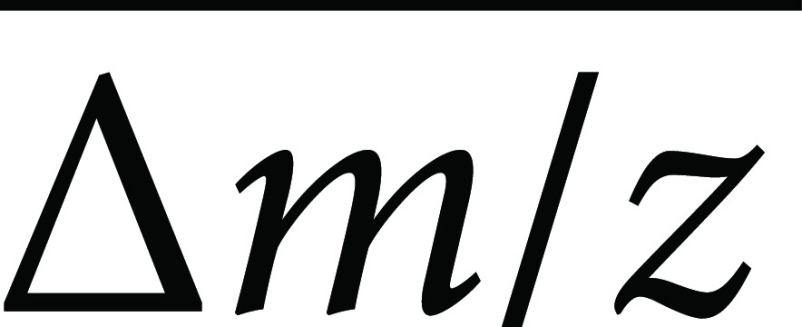
 (−0.0005) and σ (0.00203) values listed in Table 2, the range of 
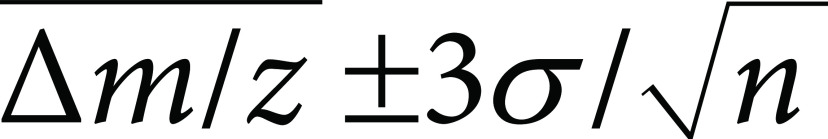
 was calculated, as shown in Fig. 3a. Nearly all of the Δ*m*/*z* values of the averaged mass spectra were within this range, indicating that the precision of *m*/*z* measurement was improved following the central limit theorem.

The same process was performed for the fragment ion data. The original and averaged product-ion spectra contained 39,011 and 616 fragment ions in total, respectively. While the standard deviation of the Δ*m*/*z* of the original data was 0.00139, that of the averaged data was 0.00042 for the averaged dataset. Moreover, Fig. 3b shows that the Δ*m*/*z* values are distributed within the range of 
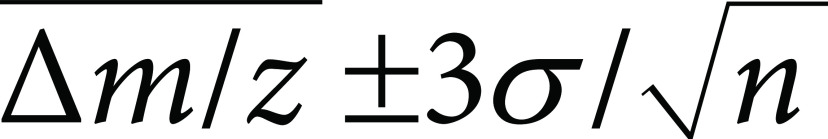
. A large standard deviation was obtained for the Δppm, as the larger Δppm values tended to be determined during the measurement of smaller *m*/*z* values included in the fragment ion data.

These results demonstrate that the Δ*m*/*z* of the level in the averaged product-ion spectra data follows the central limit theorem. This indicates that a more precise *m*/*z* value measurement can be obtained by averaging a larger number of product-ion spectra.

### Improvement of elemental composition search results using averaged data

The measured *m*/*z* values obtained from high-resolution mass spectrometry (*m*/*z*_measured_) inevitably include systematic (*e*_systematic_) and random (*e*_random_) errors, as follows: 
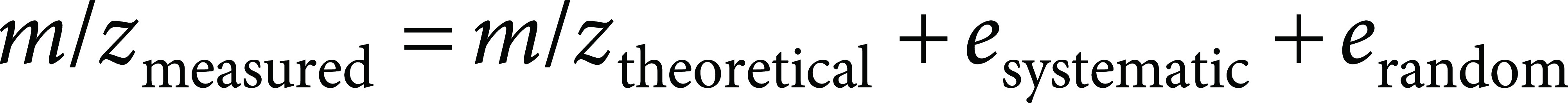
where *m*/*z*_theoretical_ represents the theoretical *m*/*z* value. The *e*_systematic_ value in this data set was deduced to be −0.80 ppm, as shown in Table 2, which would be derived from errors such as those that arose during the calibration task. The *e*_random_ values followed a normal distribution. For the case of the *m*/*z* values of precursor ions in the original dataset, the standard deviation (σ) level was deduced to be at 2.71 ppm from the Table 2. It was expected from the central limit theorem that a σ level of *e*_random_ would be 0.39 (=2.71/sqrt(50)) for averaged spectra with *n*=50.

The contribution of the smaller *e*_random_ to narrowing down the number of the candidate formula was investigated using a composition formula search. The threshold of Δ*m*/*z* was set to the 3 σ level (0.39∗3=1.17 ppm) to control the false negative rate to less than 0.23%. The Seven golden rules were employed for the composition formula search, except for rule 3 (utilization of isotopic pattern) and rule 7 (removal of chemical derivatization effect).^9)^ Rule 3 was not used because the error level in the measurement of isotopic patterns is usually larger than that required to narrow the search result.^10)^ The search ranges of the N, P, and S atom numbers were arbitrarily restricted to 0≤N≤3, 0≤P≤2, and 0 ≤S ≤1, respectively, owing to the lipidomics dataset.

**Figure 4:**
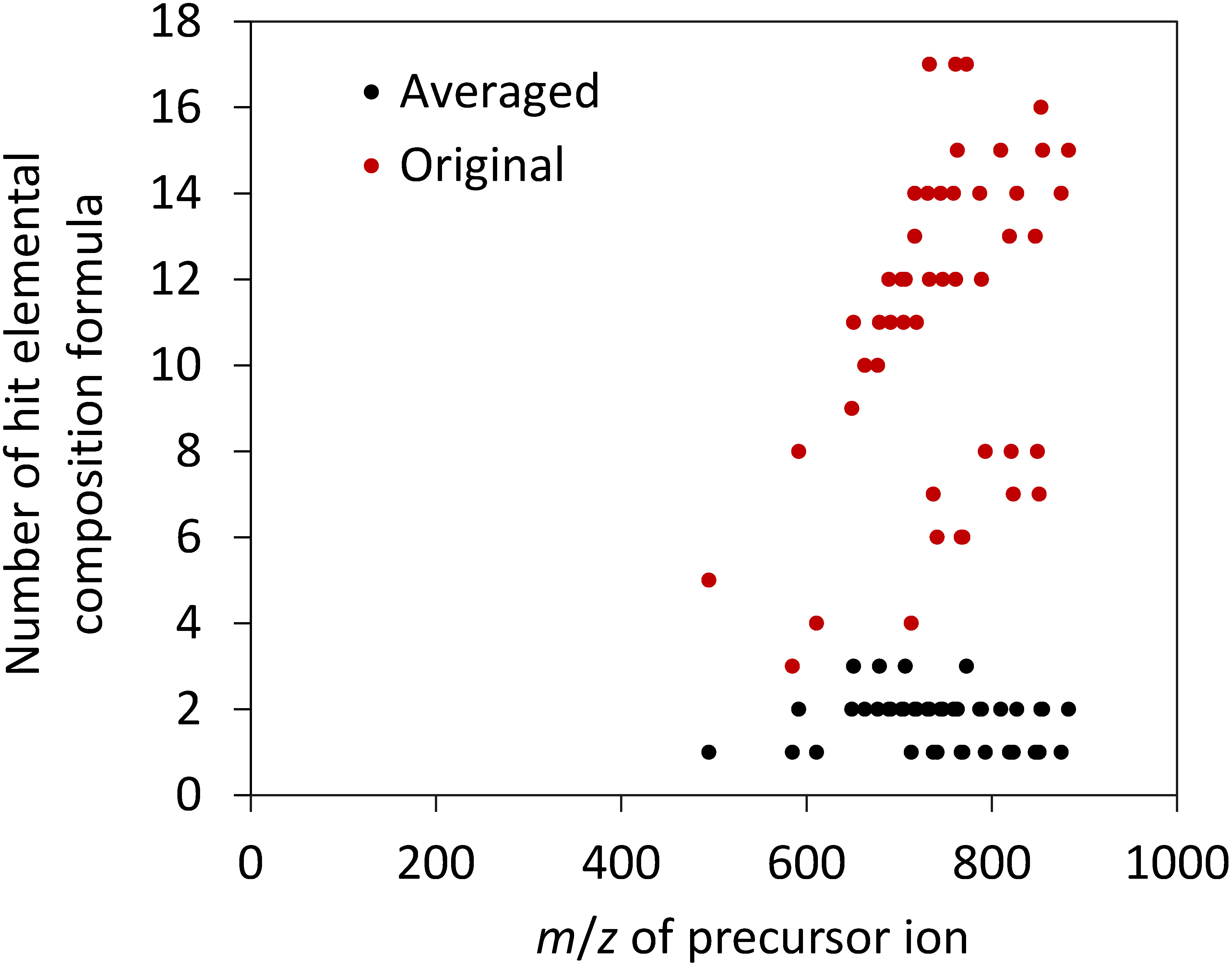
Fig. 4. Improvement of elemental composition search results by averaging. Elemental composition formula search was performed for precursor *m*/*z*_measured_ values of 48 averaged data of *n*>50 with threshold levels at 1.17 and 8.1 ppm that were suitable for elemental composition formula search of averaged (black) and original (red) data, respectively.

A composition formula search was performed for the precursor *m*/*z*_measured_ values of 48 averaged data points with *n*>50. The search results showed that only one candidate was obtained for 16 out of the 48 averaged data points (Fig. 4). The maximum and average numbers of candidates were 3 and 1.8, respectively. The composition formula search was repeated for the 48 averaged data points using a wider threshold level of (2.71∗3=) 8.1 ppm, which corresponds to the 3σ level of *e*_random_ of the original dataset before averaging. The average number of candidates increased to 11.0, which is approximately six times larger than that for the averaged data (Fig. 4). Moreover, there was a positive correlation between the number of candidates and the query *m*/*z* value. These results demonstrate that the averaged product ion spectrum data contributed to reducing the number of composition formula candidates.

## DISCUSSION

In recent years, many non-targeted metabolomics studies have employed the DDA mode to acquire product ion spectral data for metabolite annotation.^17–19,35)^ In this study, a graph-based method was applied to cluster a set of similar spectra from multiple DDA data files to create averaged product ion spectra data (Fig. 2). It was demonstrated that two averaged product ion spectra from two structural isomers (PE(34 : 1) and PC(31 : 1)) were distinctively created from a population of product-ion spectra data collected from multiple DDA data files (Table 1). This study used a yeast lipidomics dataset for proof-of-concept purposes because most of the product ions of lipids have been intensively annotated and exact answers are available.^24)^ This method does not depend on lipidomics data because averaged product ion spectra can be generated from various metabolomic DDA datasets.

The averaged spectra produced in this study are useful for the following two reasons. First, the averaged product ion spectra seem to include analyte-derived fragment ions because of reproducibility among many spectra (Fig. 1). Second, the error levels of the *m*/*z* values declined (Fig. 3 and Table 2), which allowed us to reduce the number of candidate compositional formulas (Fig. 4). This indicates that the signal-to-noise (*S*/*N*) ratio was successfully improved by averaging. The reliability and precision of the averaged spectra would contribute to more efficient annotation of the metabolite structures. However, it is inevitable that the DDA dataset will include some incorrect spectra. The method employed in this study avoids incorrect spectra by averaging the reproductively observed product ion spectra and using the medium instead of the mean value as a representative *m*/*z* value.

Moreover, the averaged spectra of the known components, such as PE(16 : 0/18 : 1) and PC(31 : 1), are also useful for enriching a spectra database as naturally derived reference data. For example, the MassBank database lacks product ion spectra data for PE(16 : 0/18 : 1) and PC(31 : 1) measured in the positive ion mode.^36)^ A set of MassBank records of averaged data created in this study (Spectrum Data 1) will be available from the data repository of MassBank Japan.

One of the challenges associated with this method is the extraction of complete graphs from similar spectral graphs. This problem is known to be one of the most computationally expensive problems (NP-Complete) and one of the most difficult to scale up.^37)^ Therefore, a heuristic approach is needed, such as hierarchical extraction using divided data rather than the batch handling of a large amount of data.^12)^ Another limitation of this method is the presence of non-isolated precursor ions. An averaged spectrum of the two metabolites was obtained for similar product ion spectra derived from two compounds with the same *m*/*z* at similar retention times. This indicates that this method can improve the signal-to-noise (*S*/*N*) ratio of the observed spectra but cannot separate the product ion spectra produced from multiple non-isolated precursor ions.

Another bottleneck to this approach is the requirement for a large number of DDA data files. However, the integration of large-scale datasets is challenging because metabolomics data repositories have been enriched in recent years.^38,39)^ If averaged spectra can be created from many DDA datasets obtained from different studies conducted at different laboratories,^40)^ it will enable a list of known and unknown metabolites to be extracted and recorded in the metabolome data.^41–43)^ Although there are issues yet to be solved, such as the standardization of data acquisition methods and the development of data analysis methods,^44)^ it is expected that the use of averaged spectra will lead to the construction of a metabolite annotation infrastructure based on actual measurement data.

## Supplementary and Spectrum Data

**Supplementary Table 1** DDA data files used in this study.

**Supplementary Table 2** Averaged product ion spectra of 3rd–9th largest cliques. The 1st and 2nd largest cliques are shown in Table 1. Estimated molecular formula and manually curated annotations were also shown.

**Supplementary Table 3** List of 100 known lipids in yeasts used to create averaged product ion spectra. The numbers of product ion spectral data used to construct the averaged spectra (*n*) as well as the MassBank record IDs of averaged data are also represented.

**Supplementary Table 4** Number of product ion spectra, total number of product ions in these spectra, and their product ion/spectra ratio in all product ion spectra around precursor *m*/*z* ±0.02, in the corresponding clique of the target lipid, and in the averaged spectra.

**Supplementary Data 1** A list of 985 Product ion spectra derived from precursor ions within *m*/*z*=718.5381±0.02 collected from the 94 DDA data files.

**Supplementary Data 2** Averaged product ion spectra of 100 known lipids in yeast.

**Spectrum Data 1** MassBank record files of averaged product ion spectra of 100 known lipids in yeast.

## Data Availability

The following data are available in J-STAGE and MassBank.

## Notes

Mass Spectrom (Tokyo) 2022; 11(1): A0106
