# Supplementary material for: Data Processing of Product Ion Spectra: Quality Improvement by Averaging Multiple Similar Spectra of Small Molecules
Source: Mass Spectrom (Tokyo). 2022 Dec 15;11(1):A0106. doi: 10.5702/massspectrometry.A0106 (PMC9853114; doi:10.5702/massspectrometry.A0106)
Supplement: Supplementary Data [file massspectrometry-11-1-A0106_s001.pdf]

Supplementary Table 1 DDA data files used in this study.

ID	DDA filename	Source
1	DDA_pos_0.*m/z*XML	Research 1: Method development
2	DDA_pos_1.*m/z*XML	Research 1: Method development
3	DDA_pos_2.*m/z*XML	Research 1: Method development
4	DDA_pos_3.*m/z*XML	Research 1: Method development
5	p_DDA_pos_0.*m/z*XML	Research 1: Method development
6	p_DDA_pos_1.*m/z*XML	Research 1: Method development
7	p_DDA_pos_2.*m/z*XML	Research 1: Method development
8	p_DDA_pos_3.*m/z*XML	Research 1: Method development
9	p_DDA_pos_4.*m/z*XML	Research 1: Method development
10	y_DDA_pos_0.*m/z*XML	Research 1: Method development
11	y_DDA_pos_1.*m/z*XML	Research 1: Method development
12	y_DDA_pos_2.*m/z*XML	Research 1: Method development
13	y_DDA_pos_3.*m/z*XML	Research 1: Method development
14	12C_0h_1_pos.*m/z*XML	Research 2: 13C-isotope labeling
15	12C_0h_2_pos.*m/z*XML	Research 2: 13C-isotope labeling
16	12C_0h_3_pos.*m/z*XML	Research 2: 13C-isotope labeling
17	12C_10h_1_pos.*m/z*XML	Research 2: 13C-isotope labeling
18	12C_10h_2_pos.*m/z*XML	Research 2: 13C-isotope labeling
19	12C_10h_3_pos.*m/z*XML	Research 2: 13C-isotope labeling
20	12C_24h_1_pos.*m/z*XML	Research 2: 13C-isotope labeling
21	12C_24h_2_pos.*m/z*XML	Research 2: 13C-isotope labeling
22	12C_24h_3_pos.*m/z*XML	Research 2: 13C-isotope labeling
23	12C_30h_1_pos.*m/z*XML	Research 2: 13C-isotope labeling
24	12C_30h_2_pos.*m/z*XML	Research 2: 13C-isotope labeling
25	12C_30h_3_pos.*m/z*XML	Research 2: 13C-isotope labeling
26	12C_3h_1_pos.*m/z*XML	Research 2: 13C-isotope labeling
27	12C_3h_2_pos.*m/z*XML	Research 2: 13C-isotope labeling
28	12C_3h_3_pos.*m/z*XML	Research 2: 13C-isotope labeling
29	12C_4h_1_pos.*m/z*XML	Research 2: 13C-isotope labeling
30	12C_4h_2_pos.*m/z*XML	Research 2: 13C-isotope labeling
31	12C_4h_3_pos.*m/z*XML	Research 2: 13C-isotope labeling
32	12C_5h_1_pos.*m/z*XML	Research 2: 13C-isotope labeling
33	12C_5h_2_pos.*m/z*XML	Research 2: 13C-isotope labeling
34	12C_5h_3_pos.*m/z*XML	Research 2: 13C-isotope labeling
35	12C_6h_1_pos.*m/z*XML	Research 2: 13C-isotope labeling
36	12C_6h_2_pos.*m/z*XML	Research 2: 13C-isotope labeling
37	12C_6h_3_pos.*m/z*XML	Research 2: 13C-isotope labeling
38	12C_8h_1_pos.*m/z*XML	Research 2: 13C-isotope labeling
39	12C_8h_2_pos.*m/z*XML	Research 2: 13C-isotope labeling
40	12C_8h_3_pos.*m/z*XML	Research 2: 13C-isotope labeling
41	wild_1_1_pos.*m/z*XML	Research 3: Strain comparison
42	wild_1_2_pos.*m/z*XML	Research 3: Strain comparison
43	wild_1_3_pos.*m/z*XML	Research 3: Strain comparison
44	wild_2_1_pos.*m/z*XML	Research 3: Strain comparison
45	wild_2_2_pos.*m/z*XML	Research 3: Strain comparison
46	wild_2_3_pos.*m/z*XML	Research 3: Strain comparison
47	wild_3_1_pos.*m/z*XML	Research 3: Strain comparison
48	wild_3_2_pos.*m/z*XML	Research 3: Strain comparison
49	0555_1_pos.*m/z*XML	Research 3: Strain comparison
50	0555_2_pos.*m/z*XML	Research 3: Strain comparison
51	0555_3_pos.*m/z*XML	Research 3: Strain comparison
52	2043_1_pos.*m/z*XML	Research 3: Strain comparison
53	2043_2_pos.*m/z*XML	Research 3: Strain comparison
54	2043_3_pos.*m/z*XML	Research 3: Strain comparison
55	B4_k7_1_pos.*m/z*XML	Research 3: Strain comparison
56	B4_k7_2_pos.*m/z*XML	Research 3: Strain comparison
57	B4_k7_3_pos.*m/z*XML	Research 3: Strain comparison

58	by1_3h_pos.*m/z*XML	Research 3: Strain comparison
59	by1_pos.*m/z*XML	Research 3: Strain comparison
60	by1_pos_pre.*m/z*XML	Research 3: Strain comparison
61	by1_pre_pos.*m/z*XML	Research 3: Strain comparison
62	by2_3h_pos.*m/z*XML	Research 3: Strain comparison
63	by2_pos.*m/z*XML	Research 3: Strain comparison
64	by3_3h_pos.*m/z*XML	Research 3: Strain comparison
65	by3_pos.*m/z*XML	Research 3: Strain comparison
66	EC1_pos.*m/z*XML	Research 3: Strain comparison
67	EC2_pos.*m/z*XML	Research 3: Strain comparison
68	EC3_pos.*m/z*XML	Research 3: Strain comparison
69	k6_1_3h_pos.*m/z*XML	Research 3: Strain comparison
70	k6_1_pos.*m/z*XML	Research 3: Strain comparison
71	k6_2_3h_pos.*m/z*XML	Research 3: Strain comparison
72	k6_2_pos.*m/z*XML	Research 3: Strain comparison
73	k6_3_3h_pos.*m/z*XML	Research 3: Strain comparison
74	k6_3_pos.*m/z*XML	Research 3: Strain comparison
75	k7_1_pos.*m/z*XML	Research 3: Strain comparison
76	k7_2_pos.*m/z*XML	Research 3: Strain comparison
77	k7_3_pos.*m/z*XML	Research 3: Strain comparison
78	k9_1_pos.*m/z*XML	Research 3: Strain comparison
79	k9_2_pos.*m/z*XML	Research 3: Strain comparison
80	k9_3_pos.*m/z*XML	Research 3: Strain comparison
81	OC1_pos.*m/z*XML	Research 3: Strain comparison
82	OC2_pos.*m/z*XML	Research 3: Strain comparison
83	OC3_pos.*m/z*XML	Research 3: Strain comparison
84	pos1.*m/z*XML	Research 3: Strain comparison
85	pos2.*m/z*XML	Research 3: Strain comparison
86	pos3.*m/z*XML	Research 3: Strain comparison
87	pos4.*m/z*XML	Research 3: Strain comparison
88	pos5.*m/z*XML	Research 3: Strain comparison
89	QA1_pos.*m/z*XML	Research 3: Strain comparison
90	QA2_pos.*m/z*XML	Research 3: Strain comparison
91	QA3_pos.*m/z*XML	Research 3: Strain comparison
92	Red1_pos.*m/z*XML	Research 3: Strain comparison
93	Red2_pos.*m/z*XML	Research 3: Strain comparison
94	Red3_pos.*m/z*XML	Research 3: Strain comparison

Supplementary Table 2 Averaged product ion spectra of 3rd – 9th largest cliques. The 1st and 2nd largest cliques were shown in Table 1. Estimated molecular formula and manually curated annotations were also shown.

	$m/z_{measured}$	Relative intensity	Estimated formula	$m/z_{theoretical}$	$\Delta m/z$	Δppm
3rd largest clique, n = 51[1)], Phosphatidyldimethylethanoamine (PDME) (32:1)						
Prec	718.5377		C39H77NO8P	718.5381	-0.0004	-0.57
Frag	170.0575	226	C4H13NO4P	170.0577	-0.0001	-0.75
	549.4879	999	C35H65O4	549.4877	0.0001	0.24
	551.4956	113	C35H68O2P	551.4951	0.0005	0.84
	718.5380	168	C39H77NO8P	718.5381	-0.0001	-0.17
4th largest clique, n = 30[1)], Remains of PE(34:1)						
Prec	718.5386		C39H77NO8P	718.5381	0.0005	0.70
Frag	577.5192	999	C37H69O4	577.5190	0.0002	0.36
	579.5266	120	C37H72O2P	579.5264	0.0002	0.30
5th largest clique, n = 16[1)], Mixture of PC(31:1) and PDME (32:1)						
Prec	718.5369		C39H77NO8P	718.5381	-0.0012	-1.73
Frag	124.9995	61	C2H6O4P	124.9998	-0.0003	-2.76
	184.0737	999	C5H15NO4P	184.0733	0.0003	1.84
	549.4803	210	C35H66O2P	549.4795	0.0008	1.53
	718.5384	376	C39H77NO8P	718.5381	0.0003	0.37
6th largest clique, n = 8[1)], Mixture of PE(34:1) and PDME (32:1)						
Prec	718.5362		C39H77NO8P	718.5381	-0.0019	-2.67
Frag	170.0583	182	C4H13NO4P	170.0577	0.0006	3.53
	549.4893	999	C35H65O4	549.4877	0.0015	2.77
	551.4956	104	C35H68O2P	551.4951	0.0005	0.87
	575.5048	84	C37H67O4	575.5034	0.0014	2.40
	577.5123	885	C37H70O2P	577.5108	0.0015	2.67
	578.5162	166	C36H68NO4	578.5143	0.0019	3.30
	718.5367	272	C39H77NO8P	718.5381	-0.0015	-2.04
7th largest clique, n = 7[1)], Remains of PE(34:1)						
Prec	718.5379		C39H77NO8P	718.5381	-0.0002	-0.26
Frag	577.5192	999	C37H69O4	577.5190	0.0002	0.32
	579.5274	128	C37H72O2P	579.5264	0.0009	1.60
8th largest clique, n = 7[1)], Remains of PC(31:1)						
Prec	718.5374		C39H77NO8P	718.5381	-0.0007	-1.00
Frag	184.0734	999	C5H15NO4P	184.0733	0.0001	0.54
	718.5395	359	C39H77NO8P	718.5381	0.0013	1.85
9th largest clique, n = 5[1)], Remains of PC(31:1)						
Prec	718.5374		C39H77NO8P	718.5381	-0.0007	-1.00
Frag	184.0734	999	C5H15NO4P	184.0733	0.0001	0.40
	718.5375	341	C39H77NO8P	718.5381	-0.0007	-0.92

1) The number of product ion spectral data used to construct the averaged spectra.

Supplementary Table 3 List of 100 known lipids in yeasts used to create averaged product ion spectra. The numbers of product ion spectral data used to construct the averaged spectra (*n*) as well as the MassBank record IDs of averaged data are also represented.

Lipid	Formula	*m/z*	Precursor	*n*[1)]	MassBank record ID[2)]
LPE(16:0)	C21H43NO7P	452.2772	$[M+H]^+$	35	MSBNK-MSSJ-OU100001
LPE(16:1)	C21H45NO7P	454.2928	$[M+H]^+$	9	MSBNK-MSSJ-OU100002
LPC(16:1/0:0)	C24H49NO7P	494.3241	$[M+H]^+$	51	MSBNK-MSSJ-OU100003
LPC(16:0)	C24H51NO7P	496.3398	$[M+H]^+$	31	MSBNK-MSSJ-OU100004
LPC(18:1/0:0)	C26H53NO7P	522.3554	$[M+H]^+$	45	MSBNK-MSSJ-OU100005
PE(28:1)	C33H65NO8P	634.4442	$[M+H]^+$	31	MSBNK-MSSJ-OU100006
PC(26:1)	C34H67NO8P	648.4599	$[M+H]^+$	64	MSBNK-MSSJ-OU100007
PC(26:0)	C34H69NO8P	650.4755	$[M+H]^+$	61	MSBNK-MSSJ-OU100008
PI(26:0)	C35H68O13P	727.4392	$[M+H]^+$	24	MSBNK-MSSJ-OU100009
PE(30:1)	C35H69NO8P	662.4755	$[M+H]^+$	50	MSBNK-MSSJ-OU100010
PC(28:1)	C36H71NO8P	676.4912	$[M+H]^+$	106	MSBNK-MSSJ-OU100011
PC(28:0)	C36H73NO8P	678.5068	$[M+H]^+$	96	MSBNK-MSSJ-OU100012
PE(32:2)	C37H71NO8P	688.4912	$[M+H]^+$	136	MSBNK-MSSJ-OU100013
PI(28:0)	C37H72O13P	755.4705	$[M+H]^+$	34	MSBNK-MSSJ-OU100014
PE(32:1)	C37H73NO8P	690.5068	$[M+H]^+$	122	MSBNK-MSSJ-OU100015
PS(32:2)	C38H71NO10P	732.4810	$[M+H]^+$	92	MSBNK-MSSJ-OU100016
PC(30:2)	C38H73NO8P	702.5068	$[M+H]^+$	104	MSBNK-MSSJ-OU100017
PC(30:1)	C38H75NO8P	704.5225	$[M+H]^+$	118	MSBNK-MSSJ-OU100018
PC(30:0)	C38H77NO8P	706.5381	$[M+H]^+$	56	MSBNK-MSSJ-OU100019
CoQ6)	C39H59O4	591.4408	$[M+H]^+$	50	MSBNK-MSSJ-OU100020
PI(30:1)	C39H74O13P	781.4862	$[M+H]^+$	27	MSBNK-MSSJ-OU100021
PC(31:2)	C39H75NO8P	716.5225	$[M+H]^+$	88	MSBNK-MSSJ-OU100022
PE(34:2)	C39H75NO8P	716.5225	$[M+H]^+$	174	MSBNK-MSSJ-OU100023
PI(30:0)	C39H76O13P	783.5018	$[M+H]^+$	19	MSBNK-MSSJ-OU100024
PC(31:1)	C39H77NO8P	718.5381	$[M+H]^+$	64	MSBNK-MSSJ-OU100025
PE(34:1)	C39H77NO8P	718.5381	$[M+H]^+$	164	MSBNK-MSSJ-OU100026
PS(34:2)	C40H75NO10P	760.5123	$[M+H]^+$	74	MSBNK-MSSJ-OU100027
PS(34:1)	C40H77NO10P	762.5280	$[M+H]^+$	81	MSBNK-MSSJ-OU100028
PC(32:2)	C40H77NO8P	730.5381	$[M+H]^+$	241	MSBNK-MSSJ-OU100029
PC(32:1)	C40H79NO8P	732.5538	$[M+H]^+$	214	MSBNK-MSSJ-OU100030
PC(32:0)	C40H81NO8P	734.5694	$[M+H]^+$	35	MSBNK-MSSJ-OU100031
PI(32:2)	C41H76O13P	807.5018	$[M+H]^+$	43	MSBNK-MSSJ-OU100032
PI(32:1)	C41H78O13P	809.5175	$[M+H]^+$	66	MSBNK-MSSJ-OU100033
PE(36:2)	C41H79NO8P	744.5538	$[M+H]^+$	100	MSBNK-MSSJ-OU100034
PC(33:2)	C41H79NO8P	744.5538	$[M+H]^+$	49	MSBNK-MSSJ-OU100035
PC(33:1)	C41H81NO8P	746.5694	$[M+H]^+$	71	MSBNK-MSSJ-OU100036
PE(36:1)	C41H81NO8P	746.5694	$[M+H]^+$	30	MSBNK-MSSJ-OU100037
PC(34:2)	C42H81NO8P	758.5694	$[M+H]^+$	279	MSBNK-MSSJ-OU100038
PC(34:1)	C42H83NO8P	760.5851	$[M+H]^+$	249	MSBNK-MSSJ-OU100039
PI(34:2)	C43H80O13P	835.5331	$[M+H]^+$	43	MSBNK-MSSJ-OU100040
PC(35:2)	C43H83NO8P	772.5851	$[M+H]^+$	28	MSBNK-MSSJ-OU100041
PC(35:1)	C43H85NO8P	774.6007	$[M+H]^+$	22	MSBNK-MSSJ-OU100042
PC(36:2)	C44H85NO8P	786.6007	$[M+H]^+$	225	MSBNK-MSSJ-OU100043
PC(36:1)	C44H87NO8P	788.6164	$[M+H]^+$	198	MSBNK-MSSJ-OU100044
Cer(44:0;3O)	C44H90NO4	696.6864	$[M+H]^+$	11	MSBNK-MSSJ-OU100045
Cer(44:0;4O)	C44H90NO5	712.6814	$[M+H]^+$	52	MSBNK-MSSJ-OU100046
PI(36:1)	C45H86O13P	865.5801	$[M+H]^+$	41	MSBNK-MSSJ-OU100047

Cer(46:0;4O)	C46H94NO5	740.7127	$[M+H]^+$	8	MSBNK-MSSJ-OU100048
PI-Cer(46:0;3O)	C52H105NO12P	966.7369	$[M+H]^+$	25	MSBNK-MSSJ-OU100049
DG(26:0)	C29H60NO5	502.4466	$[M+NH_4]^+$	5	MSBNK-MSSJ-OU100050
DG(30:1)	C33H66NO5	556.4936	$[M+NH_4]^+$	24	MSBNK-MSSJ-OU100051
DG(32:2)	C35H68NO5	582.5092	$[M+NH_4]^+$	29	MSBNK-MSSJ-OU100052
DG(32:1)	C35H70NO5	584.5249	$[M+NH_4]^+$	115	MSBNK-MSSJ-OU100053
PI(26:0)	C35H71NO13P	744.4658	$[M+NH_4]^+$	24	MSBNK-MSSJ-OU100054
DG(34:2)	C37H72NO5	610.5405	$[M+NH_4]^+$	94	MSBNK-MSSJ-OU100055
DG(34:1)	C37H74NO5	612.5562	$[M+NH_4]^+$	19	MSBNK-MSSJ-OU100056
PI(28:0)	C37H75NO13P	772.4971	$[M+NH_4]^+$	67	MSBNK-MSSJ-OU100057
DG(36:2)	C39H76NO5	638.5718	$[M+NH_4]^+$	46	MSBNK-MSSJ-OU100058
PI(30:1)	C39H77NO13P	798.5127	$[M+NH_4]^+$	24	MSBNK-MSSJ-OU100059
DG(36:1)	C39H78NO5	640.5875	$[M+NH_4]^+$	13	MSBNK-MSSJ-OU100060
PI(30:0)	C39H79NO13P	800.5284	$[M+NH_4]^+$	44	MSBNK-MSSJ-OU100061
PI(32:2)	C41H79NO13P	824.5284	$[M+NH_4]^+$	44	MSBNK-MSSJ-OU100062
PI(32:1)	C41H81NO13P	826.5440	$[M+NH_4]^+$	86	MSBNK-MSSJ-OU100063
PI(33:1)	C42H83NO13P	840.5597	$[M+NH_4]^+$	7	MSBNK-MSSJ-OU100064
TG(40:2)	C43H82NO6	708.6137	$[M+NH_4]^+$	7	MSBNK-MSSJ-OU100065
PI(34:2)	C43H83NO13P	852.5597	$[M+NH_4]^+$	50	MSBNK-MSSJ-OU100066
PI(34:1)	C43H85NO13P	854.5753	$[M+NH_4]^+$	125	MSBNK-MSSJ-OU100067
TG(42:2)	C45H86NO6	736.6450	$[M+NH_4]^+$	50	MSBNK-MSSJ-OU100068
TG(42:1)	C45H88NO6	738.6606	$[M+NH_4]^+$	19	MSBNK-MSSJ-OU100069
PI(36:1)	C45H89NO13P	882.6066	$[M+NH_4]^+$	95	MSBNK-MSSJ-OU100070
TG(42:0)	C45H90NO6	740.6763	$[M+NH_4]^+$	55	MSBNK-MSSJ-OU100071
TG(44:2)	C47H90NO6	764.6763	$[M+NH_4]^+$	45	MSBNK-MSSJ-OU100072
TG(44:1)	C47H92NO6	766.6919	$[M+NH_4]^+$	55	MSBNK-MSSJ-OU100073
DG(44:1)	C47H94NO5	752.7127	$[M+NH_4]^+$	5	MSBNK-MSSJ-OU100074
TG(44:0)	C47H94NO6	768.7076	$[M+NH_4]^+$	59	MSBNK-MSSJ-OU100075
TG(46:3)	C49H92NO6	790.6919	$[M+NH_4]^+$	19	MSBNK-MSSJ-OU100076
TG(46:2)	C49H94NO6	792.7076	$[M+NH_4]^+$	68	MSBNK-MSSJ-OU100077
TG(46:1)	C49H96NO6	794.7232	$[M+NH_4]^+$	38	MSBNK-MSSJ-OU100078
TG(46:0)	C49H98NO6	796.7389	$[M+NH_4]^+$	29	MSBNK-MSSJ-OU100079
TG(47:3)	C50H94NO6	804.7076	$[M+NH_4]^+$	22	MSBNK-MSSJ-OU100080
TG(47:2)	C50H96NO6	806.7232	$[M+NH_4]^+$	27	MSBNK-MSSJ-OU100081
TG(48:3)	C51H96NO6	818.7232	$[M+NH_4]^+$	161	MSBNK-MSSJ-OU100082
TG(48:2)	C51H98NO6	820.7389	$[M+NH_4]^+$	142	MSBNK-MSSJ-OU100083
TG(48:2;1O)	C51H98NO7	836.7338	$[M+NH_4]^+$	28	MSBNK-MSSJ-OU100084
TG(48:1)	C51H100NO6	822.7545	$[M+NH_4]^+$	108	MSBNK-MSSJ-OU100085
TG(48:0)	C51H102NO6	824.7702	$[M+NH_4]^+$	37	MSBNK-MSSJ-OU100086
TG(49:3)	C52H98NO6	832.7389	$[M+NH_4]^+$	24	MSBNK-MSSJ-OU100087
TG(49:2)	C52H100NO6	834.7545	$[M+NH_4]^+$	15	MSBNK-MSSJ-OU100088
TG(49:1)	C52H102NO6	836.7702	$[M+NH_4]^+$	13	MSBNK-MSSJ-OU100089
TG(50:1)	C53H104NO6	850.7858	$[M+NH_4]^+$	87	MSBNK-MSSJ-OU100090
TG(50:4)	C53H98NO6	844.7389	$[M+NH_4]^+$	46	MSBNK-MSSJ-OU100091
TG(50:3)	C53H100NO6	846.7545	$[M+NH_4]^+$	185	MSBNK-MSSJ-OU100092
TG(50:2)	C53H102NO6	848.7702	$[M+NH_4]^+$	160	MSBNK-MSSJ-OU100093
TG(50:2;1O)	C53H102NO7	864.7651	$[M+NH_4]^+$	10	MSBNK-MSSJ-OU100094
TG(51:2)	C54H104NO6	862.7858	$[M+NH_4]^+$	5	MSBNK-MSSJ-OU100095
TG(51:3)	C54H102NO6	860.7702	$[M+NH_4]^+$	11	MSBNK-MSSJ-OU100096
TG(52:3)	C55H104NO6	874.7858	$[M+NH_4]^+$	91	MSBNK-MSSJ-OU100097
TG(52:2)	C55H106NO6	876.8015	$[M+NH_4]^+$	8	MSBNK-MSSJ-OU100098
TG(52:2;1O)	C55H106NO7	892.7964	$[M+NH_4]^+$	5	MSBNK-MSSJ-OU100099
TG(52:4)	C55H102NO6	872.7702	$[M+NH_4]^+$	26	MSBNK-MSSJ-OU100100

1) The number of product ion spectral data used to construct the averaged spectra.
2) The MassBank record IDs are temporally.

Supplementary Table 4 Number of product ion spectra, total number of product ions in these spectra and their product ion/spectra ratio in all product ion spectra around precursor m/z ± 0.02, in the corresponding clique of target lipid, and in the averaged spectra.

		Number of product ion spectra, total number of product ions and product ion/spectra						
		Obtained from precursor *m/z* ± 0.02			in the corresponding clique of target lipid			in averaged Spectra
Target lipid	Precursor	Spectra	Product ion	Product ion/spectra	Spectra (*n*)	Product ion	Product ion/spectra	Product ion/spectra
LPE(16:0)	$[M+H]^+$	248	20439	82.4	35	2402	68.6	11
LPE(16:1)	$[M+H]^+$	161	13392	83.2	9	729	81.0	5
LPC(16:1/0:0)	$[M+H]^+$	223	19052	85.4	51	4827	94.6	14
LPC(16:0)	$[M+H]^+$	196	19967	101.9	31	2251	72.6	8
LPC(18:1/0:0)	$[M+H]^+$	171	14691	85.9	45	3687	81.9	11
PE(28:1)	$[M+H]^+$	334	24770	74.2	31	2127	68.6	2
PC(26:1)	$[M+H]^+$	183	14652	80.1	64	4286	67.0	5
PC(26:0)	$[M+H]^+$	160	13538	84.6	61	4038	66.2	5
PI(26:0)	$[M+H]^+$	100	7644	76.4	24	1293	53.9	2
PE(30:1)	$[M+H]^+$	483	38313	79.3	50	3778	75.6	3
PC(28:1)	$[M+H]^+$	343	25264	73.7	106	6566	61.9	5
PC(28:0)	$[M+H]^+$	192	14419	75.1	96	5529	57.6	6
PE(32:2)	$[M+H]^+$	353	26542	75.2	136	9130	67.1	12
PI(28:0)	$[M+H]^+$	216	15907	73.6	34	2164	63.6	2
PE(32:1)	$[M+H]^+$	898	70526	78.5	122	8751	71.7	8
PS(32:2)	$[M+H]^+$	248	16400	66.1	92	7603	82.6	3
PC(30:2)	$[M+H]^+$	447	34621	77.5	104	6418	61.7	6
PC(30:1)	$[M+H]^+$	542	39030	72.0	118	4791	40.6	7
PC(30:0)	$[M+H]^+$	208	18172	87.4	56	5024	89.7	5
CoQ6)	$[M+H]^+$	427	36964	86.6	50	6117	122.3	17
PI(30:1)	$[M+H]^+$	83	6584	79.3	27	1853	68.6	2
PC(31:2)	$[M+H]^+$	1037	75625	72.9	88	6842	77.8	5
PE(34:2)	$[M+H]^+$	1037	75625	72.9	174	9680	55.6	12
PI(30:0)	$[M+H]^+$	112	8113	72.4	19	1250	65.8	2
PC(31:1)	$[M+H]^+$	985	75678	76.8	64	4737	74.0	5
PE(34:1)	$[M+H]^+$	985	75678	76.8	164	8872	54.1	12
PS(34:2)	$[M+H]^+$	538	34436	64.0	74	4838	65.4	12
PS(34:1)	$[M+H]^+$	573	41584	72.6	81	5027	62.1	12
PC(32:2)	$[M+H]^+$	1619	117217	72.4	241	11277	46.8	7
PC(32:1)	$[M+H]^+$	1325	100915	76.2	214	9592	44.8	6
PC(32:0)	$[M+H]^+$	328	27916	85.1	35	3059	87.4	5
PI(32:2)	$[M+H]^+$	84	6991	83.2	43	3272	76.1	3
PI(32:1)	$[M+H]^+$	153	12157	79.5	66	5242	79.4	3
PE(36:2)	$[M+H]^+$	776	60868	78.4	100	7546	75.5	8
PC(33:2)	$[M+H]^+$	776	60868	78.4	49	3229	65.9	5
PC(33:1)	$[M+H]^+$	795	66358	83.5	71	5843	82.3	5
PE(36:1)	$[M+H]^+$	795	66358	83.5	30	2371	79.0	4
PC(34:2)	$[M+H]^+$	2049	139413	68.0	279	13960	50.0	6
PC(34:1)	$[M+H]^+$	1131	87867	77.7	249	10778	43.3	7
PI(34:2)	$[M+H]^+$	88	7495	85.2	43	3114	72.4	3
PC(35:2)	$[M+H]^+$	330	27980	84.8	28	1871	66.8	6
PC(35:1)	$[M+H]^+$	415	35589	85.8	22	2666	121.2	5
PC(36:2)	$[M+H]^+$	606	41780	68.9	225	8978	39.9	7
PC(36:1)	$[M+H]^+$	660	48229	73.1	198	9998	50.5	7
Cer(44:0;3O)	$[M+H]^+$	148	13578	91.7	11	1101	100.1	14
Cer(44:0;4O)	$[M+H]^+$	241	22667	94.1	52	4972	95.6	18
PI(36:1)	$[M+H]^+$	132	10797	81.8	41	3568	87.0	3
Cer(46:0;4O)	$[M+H]^+$	109	11576	106.2	8	820	102.5	19
PI-	$[M+H]^+$	77	6405	83.2	25	2142	85.7	6

Cer(46:0;3O)								
DG(26:0)	$[M+NH_4]^+$	165	13050	79.1	5	390	78.0	6
DG(30:1)	$[M+NH_4]^+$	207	16558	80.0	24	2028	84.5	12
DG(32:2)	$[M+NH_4]^+$	481	37203	77.3	29	2049	70.7	7
DG(32:1)	$[M+NH_4]^+$	625	55264	88.4	115	12923	112.4	15
PI(26:0)	$[M+NH_4]^+$	232	17080	73.6	24	1627	67.8	2
DG(34:2)	$[M+NH_4]^+$	994	82063	82.6	94	4836	51.4	5
DG(34:1)	$[M+NH_4]^+$	552	45351	82.2	19	1338	70.4	6
PI(28:0)	$[M+NH_4]^+$	102	7876	77.2	67	4893	73.0	3
DG(36:2)	$[M+NH_4]^+$	499	39728	79.6	46	4000	87.0	10
PI(30:1)	$[M+NH_4]^+$	104	8415	80.9	24	1782	74.3	5
DG(36:1)	$[M+NH_4]^+$	353	30018	85.0	13	1140	87.7	3
PI(30:0)	$[M+NH_4]^+$	93	7661	82.4	44	3180	72.3	2
PI(32:2)	$[M+NH_4]^+$	129	10292	79.8	44	3428	77.9	4
PI(32:1)	$[M+NH_4]^+$	228	19443	85.3	86	6511	75.7	3
PI(33:1)	$[M+NH_4]^+$	101	9189	91.0	7	398	56.9	2
TG(40:2)	$[M+NH_4]^+$	153	12808	83.7	7	512	73.1	10
PI(34:2)	$[M+NH_4]^+$	226	19876	87.9	50	3776	75.5	3
PI(34:1)	$[M+NH_4]^+$	375	26518	70.7	125	6840	54.7	3
TG(42:2)	$[M+NH_4]^+$	334	30373	90.9	50	4004	80.1	12
TG(42:1)	$[M+NH_4]^+$	694	56328	81.2	19	1093	57.5	10
PI(36:1)	$[M+NH_4]^+$	219	16000	73.1	95	5820	61.3	3
TG(42:0)	$[M+NH_4]^+$	330	27279	82.7	55	3496	63.6	14
TG(44:2)	$[M+NH_4]^+$	377	33205	88.1	45	4432	98.5	24
TG(44:1)	$[M+NH_4]^+$	430	38815	90.3	55	5996	109.0	40
DG(44:1)	$[M+NH_4]^+$	130	11777	90.6	5	733	146.6	14
TG(44:0)	$[M+NH_4]^+$	279	23855	85.5	59	4583	77.7	20
TG(46:3)	$[M+NH_4]^+$	239	18155	76.0	19	1616	85.1	10
TG(46:2)	$[M+NH_4]^+$	526	44464	84.5	68	5826	85.7	35
TG(46:1)	$[M+NH_4]^+$	419	40537	96.7	38	4986	131.2	34
TG(46:0)	$[M+NH_4]^+$	219	19974	91.2	29	3164	109.1	21
TG(47:3)	$[M+NH_4]^+$	104	9383	90.2	22	1901	86.4	6
TG(47:2)	$[M+NH_4]^+$	159	13406	84.3	27	2896	107.3	12
TG(48:3)	$[M+NH_4]^+$	1072	86008	80.2	161	11720	72.8	10
TG(48:2)	$[M+NH_4]^+$	1474	127045	86.2	142	10763	75.8	26
TG(48:2;1O)	$[M+NH_4]^+$	179	15456	86.3	28	1892	67.6	3
TG(48:1)	$[M+NH_4]^+$	884	80109	90.6	108	9806	90.8	20
TG(48:0)	$[M+NH_4]^+$	288	23713	82.3	37	3509	94.8	13
TG(49:3)	$[M+NH_4]^+$	188	16348	87.0	24	2356	98.2	12
TG(49:2)	$[M+NH_4]^+$	375	31438	83.8	15	1646	109.7	25
TG(49:1)	$[M+NH_4]^+$	160	14980	93.6	13	1672	128.6	18
TG(50:1)	$[M+NH_4]^+$	928	90041	97.0	87	6838	78.6	26
TG(50:4)	$[M+NH_4]^+$	86	8959	104.2	46	4195	91.2	4
TG(50:3)	$[M+NH_4]^+$	1612	134481	83.4	185	14230	76.9	20
TG(50:2)	$[M+NH_4]^+$	1634	150100	91.9	160	16449	102.8	26
TG(50:2;1O)	$[M+NH_4]^+$	207	18692	90.3	10	1103	110.3	5
TG(51:2)	$[M+NH_4]^+$	204	19432	95.3	5	450	90.0	17
TG(51:3)	$[M+NH_4]^+$	193	17853	92.5	11	1538	139.8	15
TG(52:3)	$[M+NH_4]^+$	1029	94603	91.9	91	6787	74.6	13
TG(52:2)	$[M+NH_4]^+$	1137	106364	93.5	8	634	79.3	7
TG(52:2;1O)	$[M+NH_4]^+$	185	16902	91.4	5	778	155.6	9
TG(52:4)	$[M+NH_4]^+$	145	19400	133.8	26	2623	100.9	5
Average		461.8	37645.2	83.3	66.1	4612.0	80.1	9.8
